# Efgartigimod for acute attacks of brainstem MOGAD followed by mycophenolate mofetil maintenance: a case report

**DOI:** 10.3389/fimmu.2026.1896346

**Published:** 2026-10-07

**Authors:** Fangshu Liu, Siyao Li, Miduo Tan, Zhen Zhao, Dao Zhou

**Affiliations:** 1 Department of Hematology, Zhuzhou Hospital Affiliated to Xiangya School of Medicine, Central South University, Zhuzhou, Hunan, China; 2 Department of Cardiology, Zhuzhou Hospital Affiliated to Xiangya School of Medicine, Central South University, Zhuzhou, Hunan, China; 3 Medical Center of Digestive Disease, Zhuzhou Hospital Affiliated to Xiangya School of Medicine, Central South University, Zhuzhou, Hunan, China; 4 Department of Neurology, Zhuzhou Hospital Affiliated to Xiangya School of Medicine, Central South University, Zhuzhou, Hunan, China

**Keywords:** brainstem syndrome, efgartigimod, mycophenolate mofetil, myelin oligodendrocyte glycoprotein antibody-associated disease (MOGAD), neonatal Fc receptor

## Abstract

**Background:**

Myelin oligodendrocyte glycoprotein antibody-associated disease (MOGAD) is an autoimmune inflammatory demyelinating disorder of the central nervous system with a broad clinical spectrum that includes brainstem syndromes. Evidence supporting neonatal Fc receptor (FcRn) inhibition in MOGAD remains limited.

**Case presentation:**

A 37-year-old woman presented with a MOGAD-associated brainstem syndrome characterized by diplopia, ocular-motor abnormalities, right facial weakness, and gait instability. Brain MRI demonstrated a lesion in the right middle cerebellar peduncle near the fourth ventricle. CSF analysis showed 2 leukocytes/µL, 0 erythrocytes/µL, normal protein (0.328 g/L), a type II oligoclonal-band pattern, and an elevated IgG index of 1.8 (reference value, <0.7), supporting intrathecal IgG synthesis. Serum MOG-IgG was positive by live cell-based assay. High-dose corticosteroids were discussed, but the patient declined them because of concerns regarding adverse effects. IVIG was subject to intermittent local shortage and substantial financial burden, while PLEX was considered disproportionate to the clinical severity. The patient received individualized off-label intravenous efgartigimod (20 mg/kg on Days 1 and 5) and improved clinically. After the second relapse, the same efgartigimod regimen was repeated, and low-dose oral prednisone and MMF were initiated for relapse prevention. Prednisone was discontinued approximately 5 months after initiation, whereas MMF was continued as maintenance therapy. At follow-up approximately 18 months after discharge, the patient was clinically asymptomatic.

**Discussion:**

This case documents a recognized brainstem MOGAD presentation with clinical improvement temporally associated with individualized off-label FcRn inhibition. Interpretation should consider the single-case design, absence of a comparator, rehabilitation, low-dose prednisone and MMF initiated for relapse prevention, and the natural course of the disease. This observation provides preliminary clinical context for prospective evaluation of FcRn inhibition but is insufficient to establish efficacy.

## Introduction

Myelin oligodendrocyte glycoprotein antibody-associated disease (MOGAD) is a distinct inflammatory demyelinating disorder of the central nervous system (CNS), defined by a compatible clinical phenotype and serum MOG-IgG detected using an appropriate cell-based assay (1). The recognized spectrum includes optic neuritis, acute disseminated encephalomyelitis, transverse myelitis, cerebral cortical encephalitis, and brainstem or cerebellar syndromes (2). Brainstem involvement may produce ocular-motor abnormalities, ataxia, dysarthria, or cranial-nerve-related symptoms and can create diagnostic difficulty when optic-nerve or spinal-cord manifestations are absent (3–6).

Combined gaze-palsy syndromes resulting from pontine tegmental involvement of the horizontal gaze center and the medial longitudinal fasciculus are less frequently reported in MOGAD than in multiple sclerosis. The differential diagnosis of multifocal ocular-motor abnormalities includes inflammatory disorders such as Miller Fisher syndrome and neurosarcoidosis, infectious and vascular brainstem disorders, and neuromuscular-junction disorders such as myasthenia gravis.

Established treatments for acute MOGAD attacks include high-dose corticosteroids, intravenous immunoglobulin (IVIG), and plasma exchange (PLEX) (7–9). FcRn-targeted therapies accelerate degradation of circulating IgG by blocking FcRn-mediated recycling and are being investigated in several IgG-mediated neurological diseases. Here, we report a patient with a MOGAD-associated brainstem syndrome who experienced clinical improvement temporally associated with individualized off-label efgartigimod administered for acute attack treatment rather than relapse prevention.

## Case presentation

A 37-year-old woman presented with a one-month history of right facial weakness, diplopia, and blurred vision. She was alert with fluent speech. Limb strength and tone were normal. Examination documented equal and reactive pupils, limited right-eye abduction and left-eye adduction, compensatory horizontal nystagmus on left gaze, a shallow right nasolabial fold with leftward deviation of the mouth, a midline tongue, reduced tendon reflexes, and an unsteady tandem gait; the remainder of the neurological examination was unremarkable. The combination of limited right-eye abduction, limited left-eye adduction, and compensatory horizontal nystagmus on left gaze was compatible with right internuclear ophthalmoplegia. MRI and MRA demonstrated a nodular lesion in the right middle cerebellar peduncle near the fourth ventricle, with slightly prolonged T1 and T2 signals, FLAIR hyperintensity, and no significant contrast enhancement (**Figures 1**, **2**). CSF analysis showed 2 leukocytes/µL, 0 erythrocytes/µL, protein 0.328 g/L (reference range, 0.150–0.450 g/L), glucose 3.72 mmol/L, and chloride 125.4 mmol/L. Paired CSF and serum testing demonstrated a type II oligoclonal-band pattern, defined by CSF-restricted oligoclonal IgG bands absent from serum. The CSF IgG index was elevated at 1.8 (reference value, <0.7). These findings supported intrathecal IgG synthesis but were not specific for MOGAD. Serum MOG-IgG was positive by live cell-based assay (**Figure 3A**). Together with the MRI and CSF findings, this supported a central inflammatory demyelinating process.

**Figure 1:**
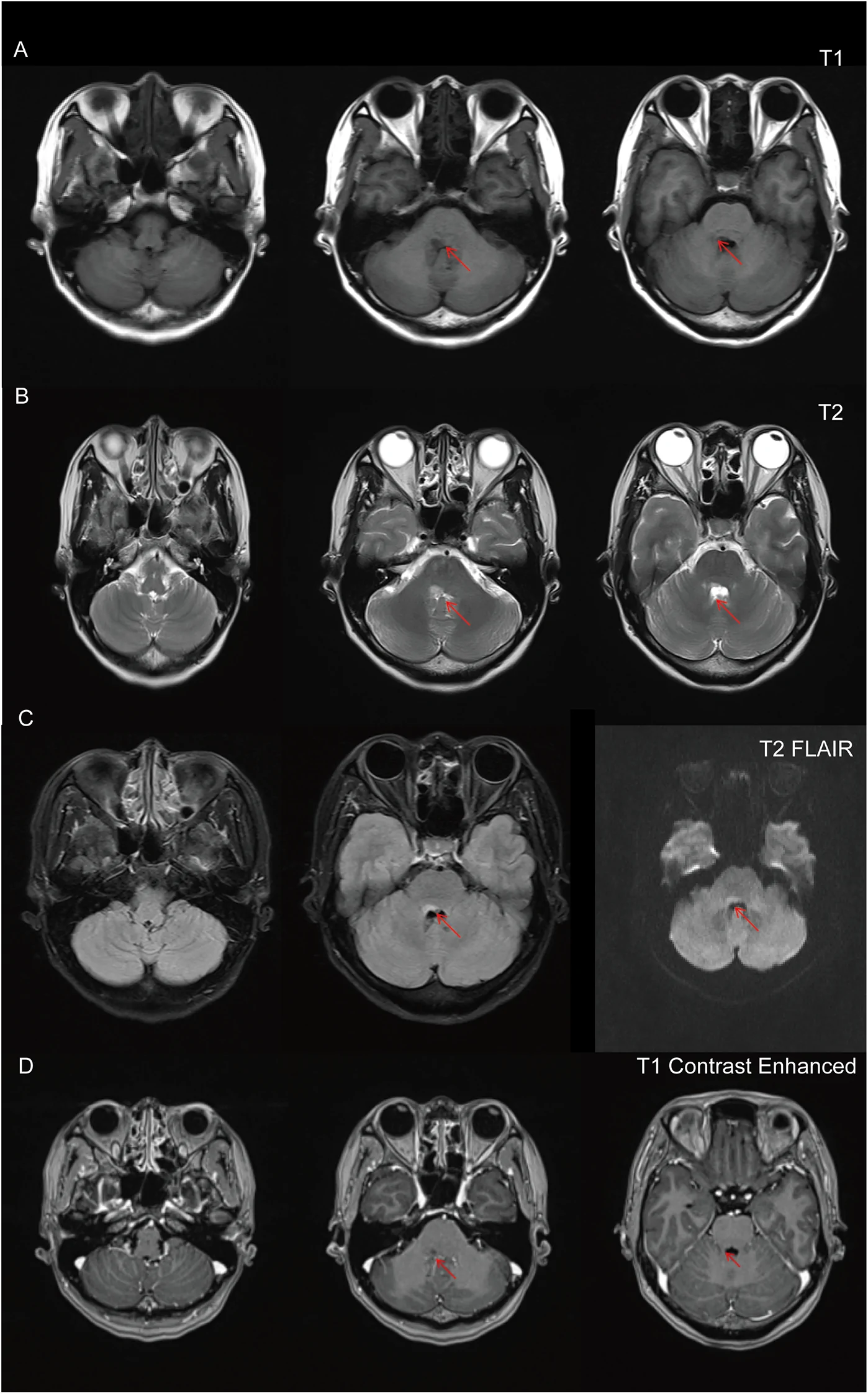
Brain MRI. **(A)** T1-weighted, **(B)** T2-weighted, and **(C)** T2-FLAIR sequences. Red arrows indicate a nodular lesion in the right middle cerebellar peduncle (MCP). **(D)** Contrast-enhanced T1 imaging showing no significant enhancement (red arrow).

**Figure 2:**
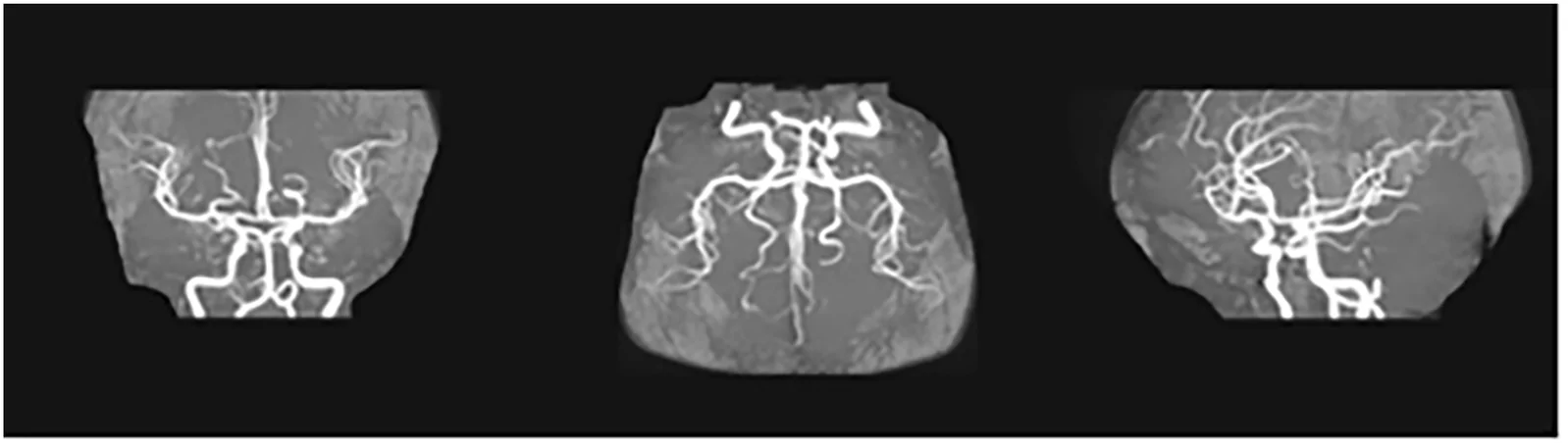
Brain MRA. MRA shows a patent left posterior communicating artery and normal intracranial vascular morphology.

High-dose corticosteroids were discussed as established first-line acute therapy; however, the patient declined high-dose treatment because of concerns regarding adverse effects. At the time, IVIG was subject to intermittent shortages and represented a substantial out-of-pocket financial burden. PLEX was considered disproportionate to the clinical severity and would have required invasive vascular access. Following multidisciplinary discussion and shared decision-making, the patient received individualized off-label intravenous efgartigimod 20 mg/kg on Days 1 and 5. Acupuncture and physical therapy were also provided. At discharge, dizziness had resolved, facial expression and ocular motility had substantially improved, and only mild limitation of right-eye abduction remained.

After the initial improvement, the patient experienced a second relapse and was readmitted because of worsening diplopia, recurrent right facial weakness, and right-sided numbness. Serum MOG-IgG was weakly positive by live cell-based assay (**Figure 3B**). Follow-up contrast-enhanced brain MRI demonstrated new patchy T1-hypointense and T2/FLAIR-hyperintense lesions in the right posterior brainstem and periventricular white matter adjacent to the trigone of the right lateral ventricle (**Figures 4A–C**). The periventricular lesion showed strip-like enhancement, whereas the posterior brainstem lesion did not enhance.

**Figure 3:**
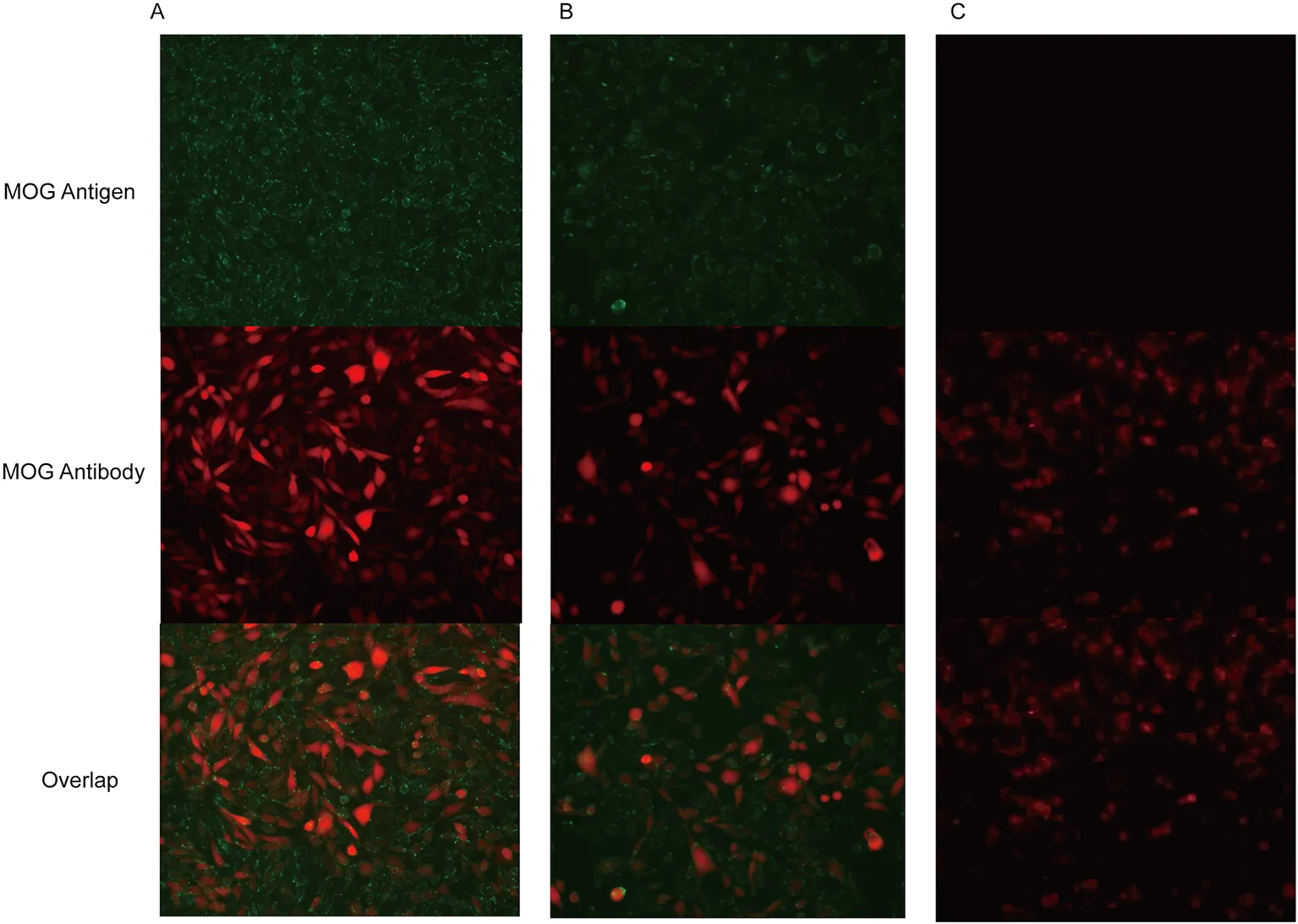
Live cell-based assay for serum MOG-IgG (Changsha ADICON Clinical Laboratories; Zhenyuan Biotech kit). Fluorescence images demonstrate MOG antigen expression (green), patient-serum IgG binding (red), and co-localization. **(A)** Initial serum testing was positive and reported semi-quantitatively as 1:32. **(B)** During evaluation of the recurrence, testing at a 1:10 serum dilution was reported semi-quantitatively as weakly positive (+). **(C)** Follow-up testing approximately seven months after symptom onset was negative.

**Figure 4:**
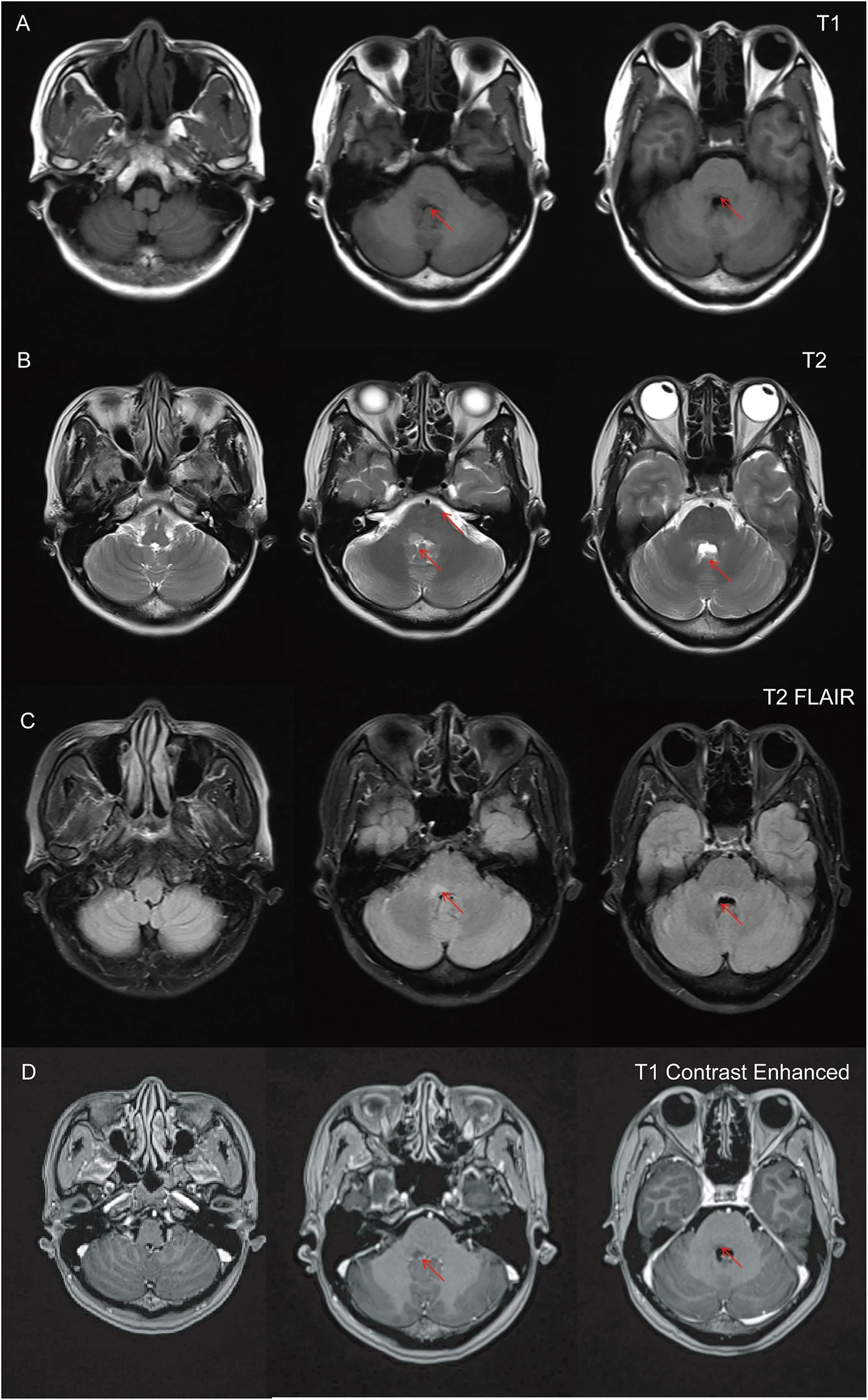
MRI. **(A)** Axial T1-weighted imaging showing new patchy hypointense lesions in the right posterior brainstem and the periventricular white matter adjacent to the trigone of the right lateral ventricle. **(B)** Axial T2-weighted imaging showing corresponding hyperintense signals in the same regions. **(C)** T2-FLAIR imaging demonstrating high-signal intensity in the right posterior brainstem and periventricular area (red arrow). **(D)** Contrast-enhanced T1-weighted imaging showed no prominent enhancement in the lesion area at the posterior region of the right brainstem (red arrow).

The individualized off-label efgartigimod regimen (20 mg/kg on Days 1 and 5) was repeated as acute treatment. Right-sided numbness began to improve, with broader clinical improvement thereafter. Low-dose oral prednisone and MMF were subsequently initiated for relapse prevention. Prednisone was prescribed at 5 mg once daily initially and 10 mg once daily at discharge. MMF was initiated at 500 mg orally twice daily and continued as long-term maintenance therapy. Efgartigimod was not used as maintenance treatment.

Following the second efgartigimod course and the initiation of low-dose prednisone and MMF, the ocular-motor abnormalities and facial weakness progressively improved. Prednisone was discontinued approximately 5 months after initiation, whereas MMF was continued for relapse prevention. This clinical course is interpreted in the context of the complete multimodal treatment and the natural course of the disease.

Follow-up serum MOG-IgG testing approximately seven months after symptom onset was negative (**Figure 3C**). At a later clinical follow-up approximately 18 months after discharge, the patient was clinically asymptomatic. MMF had been reduced from 500 mg twice daily to 250 mg twice daily and was continued at the reduced dose. The longer-term course is therefore presented in the context of ongoing MMF maintenance.

## Discussion

MOGAD is a distinct autoimmune inflammatory demyelinating disease of the CNS. Diagnostic support included serum MOG-IgG positivity by live CBA, compatible brain MRI lesions, CSF-restricted type II oligoclonal bands, and an elevated IgG index of 1.8 (reference value, <0.7). A type II oligoclonal-band pattern indicates intrathecal IgG synthesis but is not specific for MOGAD. Similarly, the elevated IgG index supports increased intrathecal IgG synthesis after correction for blood-CSF barrier permeability but does not demonstrate intrathecal synthesis of MOG-specific IgG. Serum myasthenia gravis-associated antibodies, including AChR antibodies, were negative; together with the central MRI and CSF findings, this argued against concomitant myasthenia gravis.

The rationale for FcRn inhibition in MOGAD begins with the pathogenic potential of MOG-IgG. Experimental studies have shown that patient-derived MOG-IgG can activate complement and contribute to oligodendrocyte injury and demyelination, while Fc receptor- and complement-dependent effector mechanisms both participate in MOG-antibody-mediated pathology (10). FcRn normally rescues endocytosed IgG from lysosomal degradation and returns it to the circulation. Efgartigimod competitively blocks this recycling pathway, thereby directing more IgG to lysosomal degradation and producing a rapid, selective reduction in total and pathogenic circulating IgG (11). In a MOG-IgG-augmented experimental autoimmune encephalomyelitis model, anti-FcRn treatment reduced neurological disability, spinal-cord demyelination, and macrophage infiltration and preserved visual function (12). These data provide a coherent biological rationale for FcRn inhibition in MOGAD, while remaining preclinical evidence rather than proof of clinical efficacy.

Clinical evidence for efgartigimod in MOGAD is evolving but remains preliminary. A published case of MOGAD overlapping with GFAP-IgG combined efgartigimod with corticosteroids and subsequent immunosuppression (13). A later case report described efgartigimod 10 mg/kg weekly for four doses as rescue therapy after corticosteroid and IVIG failure in isolated MOGAD (14). A small study of 27 patients with NMOSD or MOGAD compared IVMP plus efgartigimod with IVMP alone; efgartigimod was administered as 10 mg/kg for four doses or 20 mg/kg for two doses, and the add-on group showed greater short-term EDSS and IgG reductions (15).

the present report is not the first clinical use of efgartigimod in MOGAD. These reports differ from the present case, in which high-dose corticosteroids were declined but low-dose prednisone was introduced during relapse, and together provide preliminary clinical context for the potential efficacy and dosing of efgartigimod in MOGAD.

Indirect evidence also comes from other IgG-mediated neurological diseases. In the randomized phase 3 ADAPT trial in generalized myasthenia gravis, efgartigimod was generally well tolerated; treatment-emergent adverse-event and serious-adverse-event frequencies were comparable to placebo, with headache and nasopharyngitis among the most frequent events (16). In a retrospective series of five patients with myasthenic crisis, intravenous efgartigimod 20 mg/kg on Days 1 and 5 reduced total IgG and was associated predominantly with mild adverse events, although concomitant or preceding therapies and the small sample precluded causal inference (17). In a multicenter case-control study of acute anti-NMDAR encephalitis, no serious infusion-related adverse events were observed (18). Collectively, these publications provide reassurance regarding the general and short-term safety of efgartigimod. Although MOGAD-specific safety data remain limited, these findings provide relevant supportive safety context for the individualized regimen used in this case.

FcRn inhibition and therapeutic plasma exchange (PLEX) share the immediate objective of lowering pathogenic circulating antibodies during an acute attack but achieve it differently. Efgartigimod pharmacologically accelerates IgG catabolism by blocking FcRn-mediated recycling and is relatively selective for IgG (14). PLEX physically removes circulating plasma constituents, including autoantibodies, and is an established acute-treatment option in MOGAD (9). PLEX can produce rapid antibody removal but requires vascular access, specialized personnel, and replacement fluid; FcRn inhibition avoids extracorporeal circulation but depends on endogenous IgG turnover. Neither intervention directly eliminates antibody-producing B-cell or plasma-cell compartments, so antibody levels may recover after treatment. This distinction separates acute antibody-lowering treatment from long-term relapse prevention with maintenance immunotherapy such as MMF. No head-to-head study has compared FcRn inhibition with PLEX in MOGAD; the mechanistic comparison therefore informs clinical reasoning and future investigation.

The regimen used in this case was non-standard. In generalized myasthenia gravis, the ADAPT regimen comprised 10 mg/kg once weekly for four infusions (16). In published MOGAD evidence, a rescue case used 10 mg/kg weekly for four doses (14).A preliminary NMOSD/MOGAD comparative study included an intensive cohort receiving 20 mg/kg for two doses on Days 1 and 8 (15). This study provides direct clinical precedent for the 20 mg/kg dose level in an NMOSD/MOGAD acute-attack cohort, although not for the Days 1 and 5 interval used here. At the time of treatment, a registered phase 2 GBS protocol (NCT05701189) specified efgartigimod 20 mg/kg on Days 1 and 5, providing exact-schedule precedent that, together with emerging MOGAD/NMOSD experience, informed the individualized regimen used here. This schedule provides clinical context for future acute-attack dose-selection studies. No maintenance efgartigimod was administered in this case.

The acute-treatment choice requires careful contextualization. High-dose intravenous corticosteroids are an established first-line treatment for acute MOGAD attacks. The patient declined high-dose corticosteroid treatment because of concerns regarding adverse effects, although low-dose oral prednisone was subsequently introduced after the second relapse for relapse prevention. IVIG was not selected because of substantial financial burden and intermittent local shortage, whereas intravenous efgartigimod was available through the hospital pharmacy. PLEX was considered invasive relative to the clinical severity. These patient-specific considerations and local treatment availability informed shared decision-making and supported individualized off-label use of efgartigimod in this case.

The early relapse illustrates the distinction between short-term lowering of circulating IgG and long-term relapse prevention. FcRn inhibition reduces circulating IgG by preventing FcRn-mediated recycling but does not directly suppress antibody-producing B-cell or plasma-cell compartments. The 2025 French MOGAD guidelines recommend considering long-term immunoactive treatment particularly after relapse or insufficient recovery from the initial attack, following reassessment and expert-center discussion (19). Maintenance immunosuppression was not initiated after the first recognized attack because the patient had achieved substantial clinical improvement. After the early relapse with new MRI lesions and persistent MOG-IgG positivity, the risk-benefit assessment changed and MMF was initiated for relapse prevention. The subsequent relapse-free course is therefore described alongside both the preceding acute treatment and ongoing MMF maintenance.

Several considerations should be taken into account when interpreting this case. As an individual clinical observation without a comparator, the course may reflect the combined contributions of efgartigimod, rehabilitation, low-dose prednisone and MMF initiated for relapse prevention, natural recovery, and other unmeasured factors. The 20 mg/kg regimen administered on Days 1 and 5 was individualized, and no maintenance efgartigimod was administered. Accordingly, the clinical improvement cannot be attributed solely to efgartigimod.

Accordingly, this report provides preliminary clinical context for individualized efgartigimod as a potential acute-treatment option in MOGAD. The close temporal association between treatment and early improvement supports prospective evaluation of its efficacy, clinical positioning, and dosing, but the present single-case observation does not establish treatment efficacy.

Prospective studies are required to determine whether FcRn inhibition has a role in acute MOGAD, to define appropriate patient selection and dosing, and to distinguish its effects from corticosteroids, IVIG, PLEX, spontaneous recovery, and maintenance immunosuppression. Rozanolixizumab, another FcRn inhibitor, is currently being evaluated for relapse prevention in adults with relapsing MOGAD in the randomized, double-blind, placebo-controlled phase 3 cosMOG study (NCT05063162). The trial is active but no results have been posted; therefore, it provides evidence of ongoing investigation rather than established efficacy.

In conclusion, this case describes a MOGAD-associated brainstem syndrome with right internuclear ophthalmoplegia treated with individualized off-label efgartigimod during two acute attacks. Low-dose oral prednisone and MMF were initiated after the second relapse for relapse prevention, and MMF was subsequently continued as maintenance therapy. The single-case design, absence of a comparator, concomitant treatments, and natural recovery preclude causal inference. Prospective studies are required to clarify the efficacy, patient selection, and dosing of FcRn inhibition in MOGAD.

## Data Availability

The original contributions presented in the study are included in the article/supplementary material, further inquiries can be directed to the corresponding author.
